# Autochthonous and Allochthonous Gut Microbes May Work Together: Functional Insights from Farmed Gilthead Sea Bream (*Sparus aurata*)

**DOI:** 10.3390/ani16030360

**Published:** 2026-01-23

**Authors:** Alvaro Belenguer, Federico Moroni, Fernando Naya-Català, Paul George Holhorea, Ricardo Domingo-Bretón, Josep Àlvar Calduch-Giner, Jaume Pérez-Sánchez

**Affiliations:** Fish Nutrigenomics and Integrative Biology, Institute of Aquaculture Torre de la Sal (IATS, CSIC), 12595 Castellón, Spain; a.belenguer@csic.es (A.B.); fernando.naya@iats.csic.es (F.N.-C.); j.calduch@csic.es (J.À.C.-G.)

**Keywords:** gut microbiome, resident bacteria, transient bacteria, intestinal section, post-feeding time, Bayesian network, gilthead sea bream, aquaculture

## Abstract

In fish gut microbiome studies, there are no standardized protocols regarding sampling region or post-feeding time, nor clear consensus on whether analyses should target resident (autochthonous) or transient (allochthonous) bacteria. This study examined the dynamics and interactions of both microbial communities in the anterior and posterior intestine of farmed gilthead sea bream and evaluated the resident microbiome at 24 and 48 h post-feeding. Microbial DNA was sequenced using the Oxford Nanopore Technology platform. Data were analyzed through statistical and discriminant approaches, as well as a Bayesian network framework to assess bacterial interactions. Transient communities showed higher richness and diversity, regardless of intestinal section, suggesting a more specialized and stable microbial environment in the mucus layer. The two communities differed markedly in structure and composition. Variations associated with intestinal region were less pronounced, particularly for autochthonous bacteria, and post-feeding fluctuations in the resident microbiome were minimal. Functionally, results indicated relevant synergies between communities. Protein metabolism pathways were enriched in autochthonous bacteria, whereas allochthonous microorganisms contributed mainly to bile acid and carbohydrate metabolism. Overall, resident and transient bacteria constitute distinct communities in the gut of gilthead sea bream, with numerous genera present in both but most being differentially represented and interconnected.

## Simple Summary

The intestinal microbiome is highly complex in vertebrates, including farmed fish. Nevertheless, methodological approaches for its study are not standardized, and it remains unclear whether analyses should target bacteria adhered to the mucus (resident) and/or those in transit with the digesta (transient). In this study, both bacterial communities were examined in different gut sections, and resident bacteria were also assessed at various post-feeding times. Differences associated with gut region, and especially with post-feeding times for resident bacteria, were less pronounced, whereas we detected strong compositional differences between resident and transient communities. Numerous interactions between bacteria from both environments were identified. Additionally, many functional synergies appeared to occur, although each community showed specialization in several specific pathways. These results support the idea that resident and transient bacteria form distinct communities but may cooperate at a functional level, suggesting that both are relevant and should be considered separately in fish microbiome studies.

## 1. Introduction

The gastrointestinal tract represents a very dense and complex ecosystem inhabited by a large number of microorganisms that play an important role in numerous physiological processes of live animals, including fish [1,2]. Indeed, the microbiome present in the whole body, particularly in the gut systems of finfish and shellfish, has an impact on nutrition, growth, reproduction, immune system, and disease vulnerability [3,4]. Therefore, a comprehensive understanding of its structure, composition, and function is critical for elucidating the impact of microorganisms on the host. In this regard, the intestinal microbiome of fish can be shaped by numerous factors, either biotics (e.g., sex, developmental stage, fish genotype, trophic level [5,6,7]) and abiotics (e.g., rearing environment, water quality, salinity, and temperature [7,8]). Additionally, the spatial distribution across the digestive tract, the post-feeding time, and the type of bacterial community, either resident or transient, are included within those elements influencing the fish gut microbiome.

The intestinal microbiomes may differ between regions along the gastrointestinal tract, including the anterior and posterior intestine (e.g., [9,10,11]). For instance, a different composition of the microbiomes along the gut has been reported in European sea bass (pyloric caeca, midgut, and hindgut [12]). In addition, variations in the gut microbiomes among animals subjected to different post-feeding times were reported [13]. Thus, differences in taxonomic composition have been described in studies where sampling was conducted shortly after feeding (3 h; [13]) or in animals at one [14,15,16,17,18] or two [6,19,20] days post-feeding.

Concerning the type of bacteria, microorganisms in the digestive tract can be classified as transient (allochthonous) or resident (autochthonous) [21,22]. The transient bacteria are in the lumen and in transit with the intestinal contents, regarded as “short-term colonizers” that are strongly affected by environmental factors [13,23]. Conversely, the colonizing autochthonous (resident or adherent) bacteria inhabit the intestinal mucus and are considered more stable [4,24,25]. In fish microbiome studies, the selection of resident and/or transient bacterial communities for characterization has so far appeared to be based on arbitrary rather than objective criteria. Indeed, there are studies in which only one of the communities is examined [26,27] and others in which both are studied, either jointly [18,28] or independently [29,30]. In this regard, an important element to be considered may be the fish species, due to remarkable differences in the digestive anatomy. Carnivorous fish (e.g., European sea bass, gilthead sea bream) possess shorter and more resistant intestines than species with different feeding habits such as herbivores or omnivores (e.g., grey mullet, Nile tilapia), which usually present longer and thin-walled intestines [31,32]. Thus, the fragility of the gastrointestinal tract in the latter species may make it difficult to squeeze out digesta without fragmenting the intestine, which in turn may hinder the separation of transient and resident communities. In contrast, in carnivorous species, the contents of the intestinal lumen can be easily collected to study the allochthonous microbial community, and the intestinal mucus is then washed with sterile saline buffer before taking mucus scrapes or swabs to investigate the autochthonous microbial community [22,33,34].

Overall, for fish gut microbiome studies, there are neither standard protocols for sampling nor a clear consensus on which bacterial community should be targeted. Despite this, it is generally accepted that the mucus colonizing autochthonous bacteria are more stable and have a more direct impact on fish physiology [35]. In contrast, the allochthonous bacteria seem to have a more transient effect [36], although their interconnection with the host metabolism may also be relevant [13]. In fact, many probiotics, defined as “live microorganisms that confer a health benefit on the host when administered in adequate amounts” [37], appear to exert a positive effect as part of the transient bacterial community, without necessarily colonizing the intestinal mucus [38,39]. Nonetheless, the organization of both communities, together with their functional role, still remains far from being clear. Accordingly, this study aimed to assess how the gut autochthonous and allochthonous bacterial communities change and interact in a spatial (different sections of the gut) and temporal (varying post-prandial times) manner in farmed fish. To that purpose, the emerging Oxford Nanopore Technology platform was employed for DNA sequencing as an accurate and efficient approach for rapidly profiling diverse aquaculture microbiomes [40], using gilthead sea bream as a representative farmed fish in the Mediterranean aquaculture due to its high production volume and economic importance [41].

## 2. Materials and Methods

### 2.1. Ethics Statement

All the procedures received approval from the Ethics and Animal Welfare Committee of the Institute of Aquaculture Torre de la Sal (IATS), the CSIC Ethics Committee (with the authorization number 1295/2022), and the Generalitat Valenciana (under the licence number 2022-VSC-PEA-0230). These procedures were conducted at the registered aquaculture infrastructure facility of IATS (facility code ES120330001055), adhering strictly to the guidelines set forth in the European Animal Directive (2010/63/EU) and the Spanish legal framework (Royal Decree RD53/2013), for the protection of animals used in scientific experiments.

### 2.2. Animals and Sample Collection

A total of 90 two-year-old gilthead sea bream of Mediterranean origin (Avramar, Burriana, Spain) were grown in 3000 L tanks from the early life stages in a flow-through system, ensuring the oxygen content of water effluents was above 75% saturation, under natural photoperiod and temperature conditions at the IATS aquaculture infrastructure (latitude 40°5′ N; 0°10′ E). Animals were fed daily (once or twice a day depending on fish size and season) with automated feeders near to visual satiety with a standard commercial diet (INTRO Plus MT or EFICO 3053, Biomar, Palencia, Spain). In April 2024, when the water temperature was approximately 16–18 °C and the fish had reached an average body weight of about 350 g (356 ± 12.8 g) and a stocking density of around 10–11 kg/m^3^, 10 animals, fed once daily (9:30 pm), were anesthetized with 0.1 g/L tricaine methanesulfonate (Tricaine Pharmaq, PharmaQ AS, Overhalla, Norway) following a 24 h post-feeding period. From these 10 fish, we simultaneously collected mucus and content samples from the anterior and posterior intestine for the analysis of autochthonous and allochthonous bacteria, respectively. At 48 h post-feeding time, 10 other fish were anesthetized to obtain only intestinal mucus samples, as no digesta remained within the intestinal lumen. For both samplings, after dissecting the digestive system, the intestine (excluding the pyloric caeca) was divided into anterior and posterior sections. The contents of each section were then obtained by squeezing and stored in sterile tubes to collect samples of the transient intestinal microbiome. Afterwards, both sections were opened and washed with sterile Hank’s balanced salt solution before the scraping of mucus in a portion of the anterior and posterior intestine with the blunt edge of a sterile scalpel to collect the resident intestinal microorganisms. Samples of intestinal mucus were transferred to sterile tubes and kept on ice until DNA extraction, which was performed immediately after sampling to minimize post-mortem microbial shifts and host tissue degradation. In contrast, samples corresponding to the transient (luminal) intestinal microbiome were snap-frozen and stored at −80 °C until DNA extraction, as freezing effectively preserves microbial DNA integrity for downstream analyses. No chemical fixatives (e.g., ethanol or RNAlater) were used.

### 2.3. Bacterial DNA Extraction

DNA from transient intestinal microbiome was obtained using the purification DNeasy PowerSoil Pro Kit (Qiagen, Hilden, Germany). First, intestine samples (approximately 150–200 mg) were diluted with lysis buffer and submitted to a mechanical lysis using the ceramic bead tubes provided in the kit, using the FastPrep 24 homogenizer (MP Biomedicals, Irvine, CA, USA) at 6 m/s for 30 s. Subsequent steps of the extraction were performed following the manufacturer’s instructions. DNA from resident intestinal mucus microbiome (200 µL) was extracted using the High Pure PCR Template Preparation Kit (Roche, Basel, Switzerland) following the manufacturer’s recommendations, including a previous lysis step with lysozyme (Sigma, Darmstadt, Germany) at a concentration of 250 µg/mL for 15 min at 37 °C [6]. DNA concentration and quality were checked in both cases using NanoDrop 2000c (Thermo Fisher Scientific, Waltham, MA, USA) and agarose gel electrophoresis (1% *w*/*v* Tris-EDTA buffer). All extracted DNA samples were stored at −20 °C until sequencing.

### 2.4. Oxford Nanopore Technologies MinION Sequencing of Transient and Resident Microbiome

To characterize the microbiome of the intestinal contents and mucus, the Oxford Nanopore Technologies (ONT, Oxford, UK) MinION sequencing platform was employed. To do so, the complete V1–V9 region of the bacterial 16S rRNA gene was amplified and barcoded from the DNA samples using the Native Barcoding Kit 96 V14 (LIG; SQK-NBD114.96) together with 27F–1492R barcoded primers and adapted PCR conditions [40]. All PCRs were conducted in a total volume of 25 µL; 12.5 µL of LongAmp Hot Start Taq 2× Master Mix (New England Biolaps, Ipswich, MA, USA), 1 µL of each primer (9 µM), and 10.5 µL of template DNA at the corresponding concentration for each type of sample made up with Ultrapure DNase/RNase-Free Distilled Water (Invitrogen, Waltham, MA, USA). In order to check for possible contamination, negative controls were added. After a clean-up step using Agencourt AMPure XP Beads (Beckman Coulter, Brea, CA, USA), using a beads/sample ration of 0.4, amplicons were visualized in agarose gel (1% *w*/*v* TAE buffer) to ensure the presence of the specific band of ≈1500 bp, and DNA concentrations were quantified by fluorescence using PicoGreen dye (Thermo Fisher, Waltham, MA, USA). A total of 40 libraries were sequenced in MinION devices using R. 10.4.1 flow cell after flushing it with the Flow Cell Wash Kit (EXP-WSH004, ONT, Oxford, UK), always employing unique barcodes in each flow cell to prevent cross contamination between subsequent runs. Libraries were built at a reason of one individual per sample (6 samples for resident bacteria of the anterior intestine after 24 h post-feeding, Rd-AI1; 8 for resident bacteria of the anterior intestine after 48 h post-feeding, Rd-AI2; 6 for resident bacteria of the posterior intestine after 24 h post-feeding, Rd-PI1; 8 for resident bacteria of the posterior intestine after 48 h post-feeding, Rd-PI2; 6 for transient bacteria of the anterior intestine after 24 h post-feeding, T-AI; and 6 for transient bacteria of the posterior intestine after 24 h post-feeding, T-PI).

Sequencing data were acquired using MinKNOW v24.02.6 software. Raw sequencing POD5 files from the runs in this work were basecalled using Dorado v0.7 (https://github.com/nanoporetech/dorado; accessed on 25 June 2025) with a computer equipped with an Nvidia RTX 4009 24 GB GPU. The high accuracy basecalling algorithm (HAC) was applied to all the sequenced samples, as described by [40]. Then, the basecalled samples were demultiplexed and trimmed from barcodes and adapters using Dorado v0.7, and the resulting BAM files were converted into FASTQ format using samtools v1.10 [42]. These obtained raw sequenced data were lodged in the Sequence Read Archive (SRA) under the Bioproject accession number PRJNA1372998 (BioSample accession numbers: SAMN53634082-121). The resulting FASTQ files were pre-processed using Chopper v0.8.0 [43]. Samples were filtered for quality using a minimum threshold (q = 11). Quality and length metrics were obtained for each sample using NanoPlot v1.42.0 [43]. Filter reads were then taxonomically assigned with minimap2 v2.28-r1209 [44] using SILVA v138.1 as a reference database [45].

### 2.5. Statistical and Data Analysis

Normality of the data was verified by Shapiro–Wilk test. Rarefaction curves, species richness estimates, and alpha diversity indices were obtained using phyloseq package for R v4.2.2 [46]. Statistical differences in species richness, alpha diversity indices, and bacterial relative abundances were determined by Kruskal–Wallis test using Dunn’s post-test, with a significance threshold of *p* < 0.05. To study detailed microbiome differences among groups, partial least-squares discriminant analyses (PLS-DA) were performed using EZinfo v3.0 (Umetrics, Umeå, Sweden). The quality of the PLS-DA model was evaluated by the parameters R2Y (cum) and Q2 (cum), which indicate the model fit and prediction ability, respectively. The contribution to group separation of the different bacterial genera was determined by the variable importance in projection (VIP) value. VIP score > 1 was considered the threshold level to determine discriminant variables in the PLS-DA model [47,48].

To study the intestinal bacterial interactions within the microbiome populations, a stochastic model, based on the construction of a comprehensive Bayesian network (BN), was applied. For this purpose, the bacterial relative abundances at genus level of the autochthonous and allochthonous microbial communities were combined and considered as input dataset for microbiome, while the type of bacterial community (autochthonous or allochthonous) was used as discrete experimental variable. The BN was built under the following parameters: bacterial taxa were normalized using the equation described in Moroni et al. [49]; taxa with zero total counts of normalized data were removed; and to fit the model, the Zero-inflated Negative Binomial (ZINB) distribution of the normalized microbial abundances was used and the strength of each connection (edge) in the model was calculated using Bayesian information criterion (BIC) and mutual information (MI) criterion, fixing the threshold at MI < 0.05 and BIC < 0. This model allowed the identification of the causal relationships between the variable and microbial taxa, defining a network hierarchy, by which the probabilistic dependence of one node to another is defined through a parent–child relationship. The build of the BN was performed using the on-line SAMBA tool (Structure-learning of aquaculture microbiomes using a Bayesian approach) V2 already described [50]. This software makes feasible to identify clusters of nodes (bacteria) densely connected to each other, using the Leiden community detection method [51]. The resulting clusters were then used to conduct inferred metagenome analysis using PICRUSt2 protocol, assigning metagenomic pathways with the Kyoto Encyclopedia of Genes and Genomes (KEGG) [52]. Raw KEGG pathway data was then normalized and analyzed using the Kruskal–Wallis test with a significance threshold of *p* < 0.05.

## 3. Results

### 3.1. Richness and Diversity of Gut Microbiome

**Figure 1:**
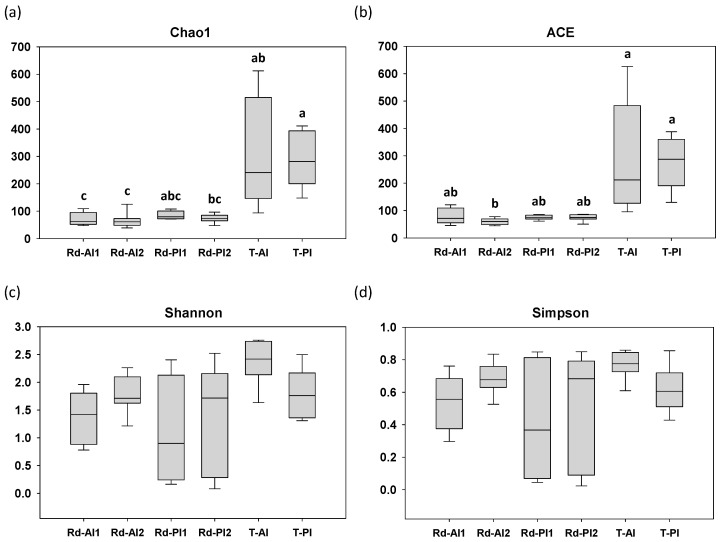
Boxplots representing the richness estimators (**a**) Chao1 and (**b**) ACE, and the diversity indices (**c**) Shannon and (**d**) Simpson of the resident (Rd) and transient (T) microbiomes of anterior (AI) and posterior (PI) intestine after 24 (1) or 48 h (2) post-feeding (n = 6 for samples collected after 24 h post-feeding and n = 8 for those collected at 48 h post-feeding time). Different letters indicate significant differences among sample types (Kruskal–Wallis test with Dunn’s post-test, *p* < 0.05).

A total of 958,619 high-quality assigned reads for 40 gut samples were obtained, with an average of 23,965 reads per sample. The high percentages of these reads were classified up to genus (average of 73.6%), family (average of 95.8%), and phylum (average of 99.9%) levels. Figure 1 and Table S1 show the richness (Chao1 and abundant-based coverage estimator, ACE) and diversity (Shannon and Simpson) indices for the six types of samples (Rd-AI1, Rd-AI2, Rd-PI1, Rd-PI2, T-AI, T-PI). Concerning the Chao1 index, the transient bacteria samples, either from the anterior or posterior intestine, showed greater values of richness in comparison to samples of the resident bacteria, regardless of the sampled intestinal section and the post-feeding time (Figure 1a and Table S1), although when all types of samples were compared, differences were not always statistically significant due to the high individual variability. Similar results were obtained for ACE (Figure 1b). In contrast, no significant differences in diversity indices (i.e., Shannon and Simpson) were observed among all sample types (Figure 1c,d). Furthermore, statistical analysis of both diversity indices between the autochthonous and allochthonous bacterial communities at 24 h post-feeding displayed significant differences (Table S1). No significant differences were detected between the anterior and posterior intestinal sections at 24 h post-feeding, either for the resident or the transient communities, nor within the resident community between 24 and 48 h post-feeding.

### 3.2. Gut Microbiome Composition

**Figure 2:**
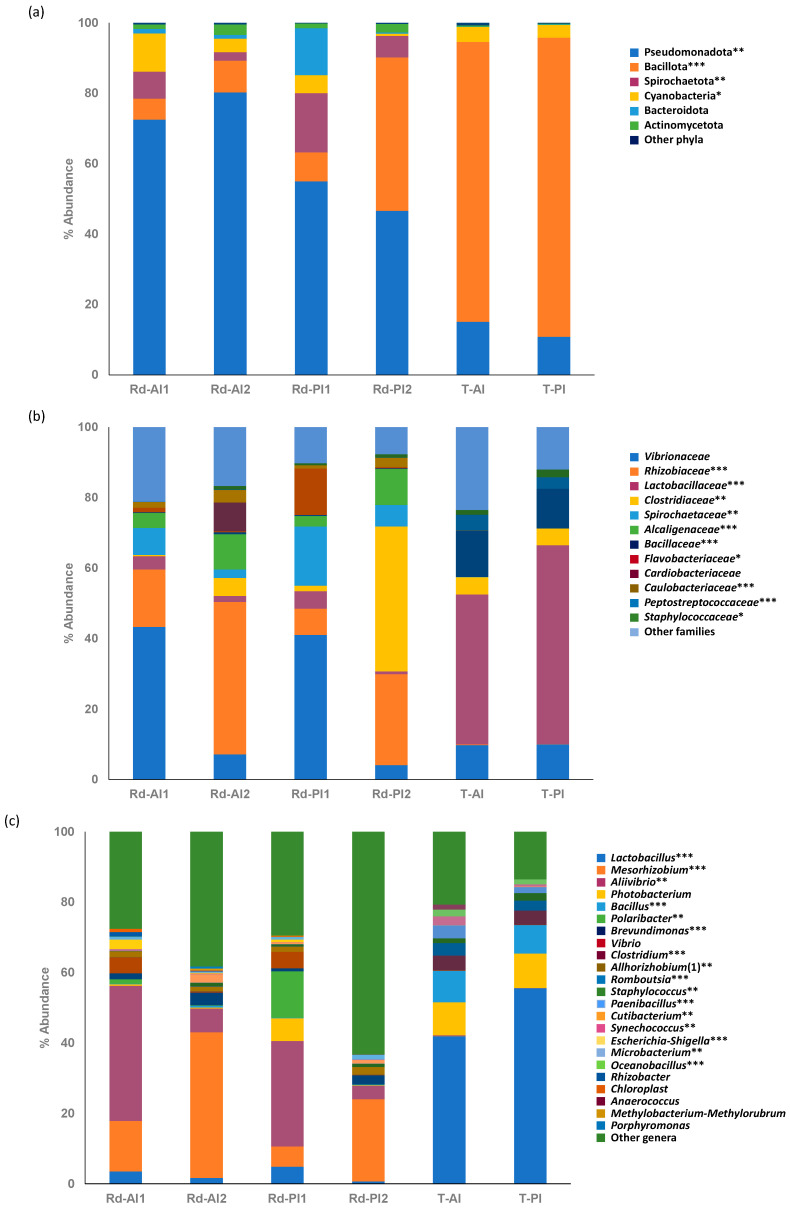
Stacked bar chart representing the relative abundance of (**a**) bacterial phyla, (**b**) family, and (**c**) genera for the resident (Rd) and transient (T) microbiomes of anterior (AI) and posterior (PI) intestine after 24 (1) or 48 h (2) post-feeding (n = 6 for samples collected after 24 h post-feeding and n = 8 for those collected at 48 h post-feeding time). Asterisks indicate the statistical significance of the Kruskal–Wallis analysis (*, *p* < 0.05; **, *p* < 0.01; ***, *p* < 0.001) conducted to compare the six experimental groups. Dunn’s post-test was performed for a multiple comparison, and the results can be accessed at Table S2. (1) Refers to *Allhorizhobium-Neorhizobium-Pararhizobium-Rhizobium*.

Gut microbiome composition at the phylum, family, and genus levels are displayed in Figure 2. Resident bacterial communities (Rd-AI1, Rd-AI2, Rd-PI1, Rd-PI2) were characterized by a predominance of the Pseudomonadota phylum, whereas Bacillota was the highest represented taxa in the transient bacteria (T-AI, T-PI; Figure 2a). In any case, a great individual variability was observed, and differences did not always reach statistical significance (Table S1). Other relevant phyla were Spirochaetota, Cyanobacteria, Bacteroidota, and Actinomycetota in the autochthonous bacteria, and Cyanobacteria in the allochthonous microbiome. Together, these phyla accounted for approximately 19% of the total bacteria in the resident community and 4% in the transient community, respectively.

Despite the lower number of phyla with relevant abundance in the transient bacteria (as shown in Figure 2a), a greater number of families (Figure 2b) and specially genera (Figure 2c) were detected within the samples of this allochthonous community. Concerning the families, *Vibrionaceae*, *Rhizobiaceae*, *Spirochaetaceae*, *Alcaligenaceae*, and *Caulobacteriaceae* showed higher abundances in the resident microbiome, whereas *Clostridiaceae*, *Bacillaceae*, *Lactobacillaceae*, *Peptostreptocacceae* and *Staphylococcaceae* were more abundant in the transient microbiome (Figure 2b and Table S2). It is noteworthy that most bacteria of the phylum Bacillota in the allochthonous bacterial community belonged to the family *Lactobacillaceae* and the genera *Lactobacillus*, and both represented in average over 40% of the total transient community either in the anterior or the posterior intestine (Table S2). In contrast, the most prevalent families and genera of the phylum Pseudomonadota within the autochthonous bacteria were *Rhizobiaceae*, including the genus *Mesorhizobium*, and *Vibrionaceae*, with *Aliivibrio*. On average, these genera accounted for 21.2% (*Mesorhizobium*) and 19.7% (*Aliivibrio*) of the total autochthonous microbial community.

### 3.3. Discriminant Analysis

In order to compare the microbiomes of the resident bacterial community at 24 and 48 h post-feeding, a PLS-DA model was constructed. Regarding the temporal differences (24 vs. 48 h), the results indicated no clear separation between the two profiles, as the validation of the PLS-DA did not reach significant values (Figure S1a,b). A similar analysis was performed to investigate differences in the bacterial composition of the autochthonous and allochthonous communities in the anterior and posterior sections of the intestine. In this case, the statistical validated model revealed three separate groups, resident microbiome (Rd-AI1 + Rd-PI1), transient microbiome of the anterior intestine (T-AI) and transient microbiome of the posterior intestine (T-PI; Figure S2a,b). Thus, certain variations appeared in the allochthonous bacterial communities between both sections of the gut. Nonetheless, these changes were due to effects on minor bacterial groups with low abundance (<0.15%), as displayed in the loading plot (genera *Atopococcus*, *Soenhgenia*, *Vagococcus*, *Brevibacterium*, *Planctomicrobium*, *Tropicibacter*, *Rhodopirellula*, and *Blastopirellula*, and families *Methyloligellaceae* and *Gimesiaceae*; Figure S2c).

**Figure 3:**
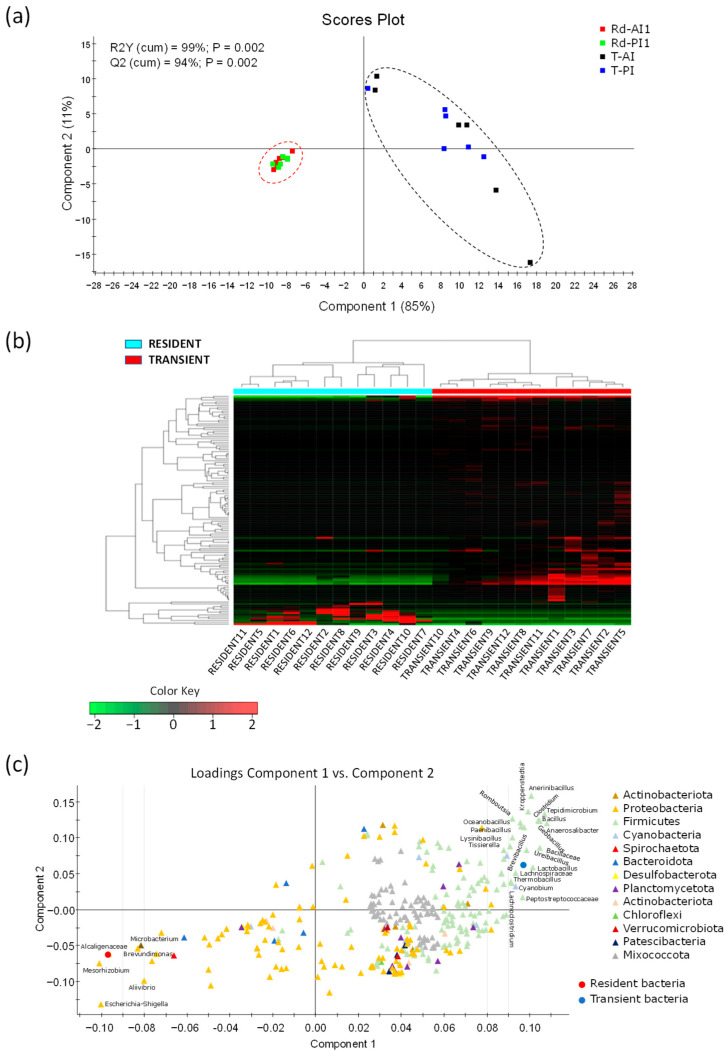
(**a**) PLS-DA Score plot showing the distribution of the samples between the first two components in the model driving the separation of the resident (Rd, from anterior, AI, or posterior, PI, intestine) and transient (T, from AI or PI) bacteria at 24 h post-feeding. (**b**) Heatmap representing the abundance distribution (*Z-*score) of the OTUs driving the separation between the gut resident and transient bacteria at 24 h post-feeding, regardless of the sampled intestinal section. Rd and T microbiomes are represented in blue and red, respectively. (**c**) Loading plot of the ecological grouping in the PLS-DA model shown in (**a**), reporting the main taxa markers associated with each type of bacterial community, resident (red circle) and transient (blue circle), indicating the phylum for each taxon.

When samples of the resident and transient bacteria collected at 24 h post-feeding were compared regardless of the intestinal section, the discriminant analysis displayed a clear and significant separation (Figure 3), with approximately 96% of the total variance being explained by the two first components (Figure 3a). Figure S3 shows the validation of the model by random permutation. In this case, the separation was further confirmed by the heatmap, where all samples were correctly classified by the hierarchical clustering in their corresponding group (Figure 3b). Additionally, the loading plot (Figure 3c) supported the strong differences in bacterial composition between both communities, since it allowed to identify the main taxa associated with each community, being mostly of the phylum Pseudomonadota in the resident community and of Bacillota in the transient bacteria.

**Figure 4:**
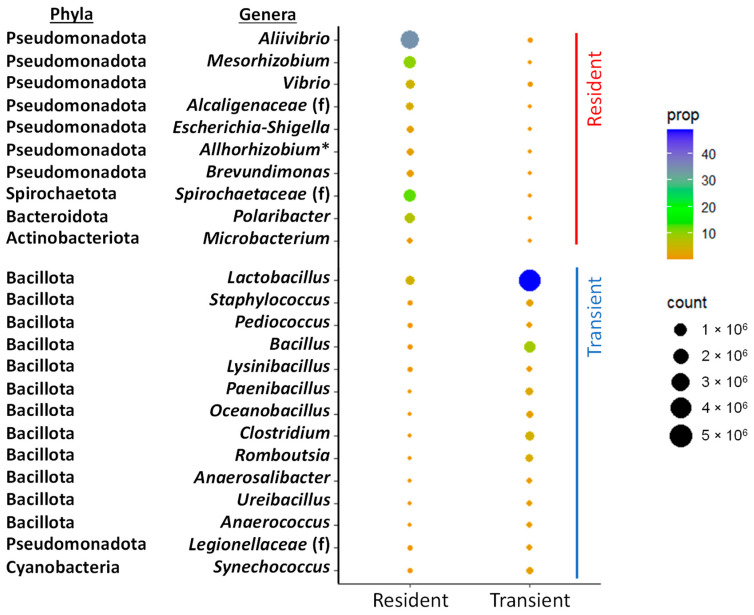
Dot plot representing discriminant taxa (VIP > 1) with more than 0.5% relative abundance in at least one microbial community. “Resident” refers to the autochthonous microbiota associated with the intestinal mucus, whereas “Transient” refers to the allochthonous microbiota corresponding to the intestinal lumen content. The colour scale represents the mean relative abundance (%) of each taxon within each group, and the size of the dots represents normalized counts. (*) Asterisk indicates that the complete taxa name is *Allorhizobium–Neorhizobium–Pararhizobium–Rhizobium*; (f) indicates unclassified members of the corresponding family.

To better assess genus-level abundance patterns and identify the specific bacterial taxa driving differences between the two community types, only genera with a relative abundance above 0.5% and a VIP value ≥ 1 in the discriminant analysis were considered. This approach led to the identification of 24 taxa considered responsible for distinguishing resident from transient bacterial communities (Figure 4). Of these, 10 were more abundant in the resident community (*Aliivibrio*, *Mesorhizobium*, *Vibrio*, *Escherichia-Shigella*, *Allorhizobium-Neorhizobium-Pararhizobium-Rhizobium*, *Brevundimonas*, *Polaribacter*, and *Microbacterium* genera; *Alcaligenaceae* and *Spirochaetaceae* families), while 14 were more abundant in the transient community (*Lactobacillus*, *Staphylococcus*, *Pediococcus*, *Bacillus*, *Lysinibacillus*, *Paenibacillus*, *Oceanobacillus*, *Clostridium*, *Romboutsia*, *Anaerosalibacter*, *Ureibacillus*, *Anaerococcus*, and *Synechococcus* genera; *Legionellaceae* family).

### 3.4. Bayesian Network and Functional Profiles of Gut Microbiome

**Figure 5:**
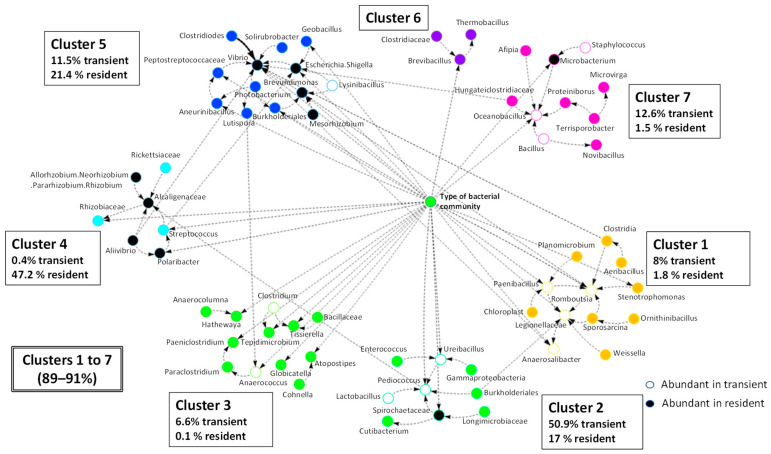
Bayesian networks representing the resident and transient bacteria model. Circles represent bacterial taxa, except the green circle in the middle that represents the experimental variable (type of bacterial community). The range of relative abundances of the taxa in clusters 1–7 is shown, together with those of the bacteria sampled from the resident or transient bacteria in each cluster. White and black circles indicate taxa presenting a significantly greater relative abundance in the transient and resident microbiomes, respectively.

To construct the BN, we used data from the resident and transient bacterial communities 24 h after the last feeding regardless of the intestinal section. The resulting model included 123 nodes, which covered 99.0% and 98.0% of the total average abundances of the microbial population for the autochthonous and allochthonous communities, respectively. A Leiden hierarchical clustering was applied, which allowed for the identification of up to 7 clusters directly connected with the experimental variable (Figure 5). This interconnected network comprised 66 nodes, representing 89.8% and 91.0% of the abundance in the resident and transient bacterial communities, respectively. Notably, it included 23 of the 24 taxa previously identified (Figure 4) as key drivers distinguishing the two communities. Moreover, clusters 4 and 5 presented a greater contribution of the resident bacteria, whereas clusters 2 and 7 displayed a dominance of microorganisms of the lumen layer.

**Figure 6:**
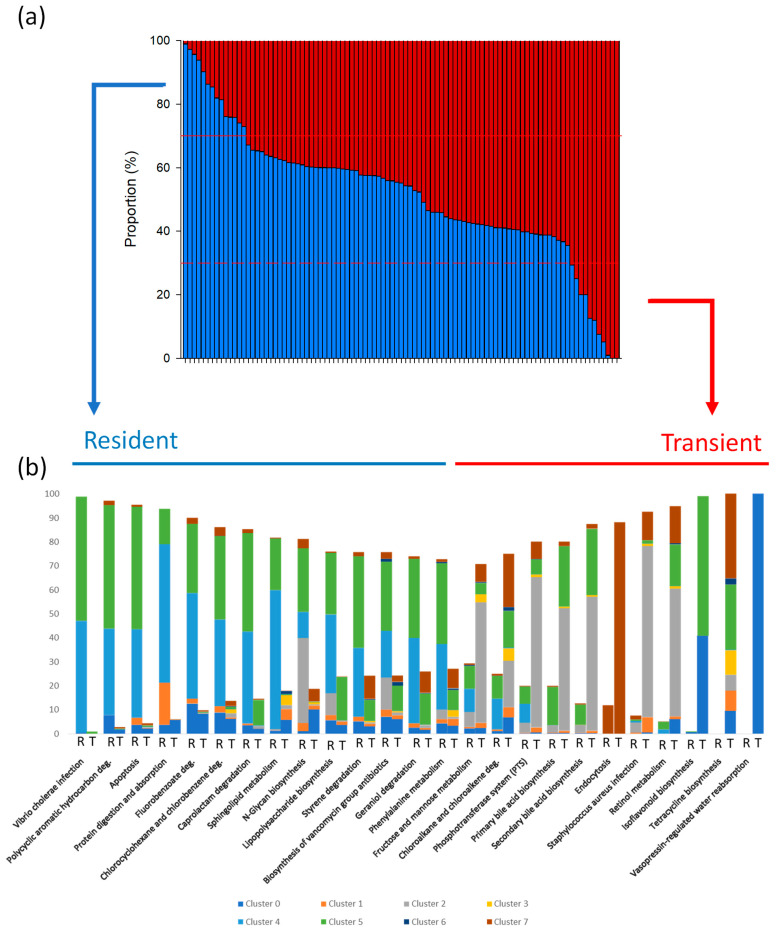
(**a**) Diagram reporting the contribution of each type of bacterial community, resident and transient, in the significant (*p*-adjusted < 0.05) functional enrichments obtained for both communities. The red lines indicate the 70% contribution of the resident (continuous line) and transient (dashed line) bacterial communities to the enriched pathways. (**b**). Contribution of each cluster of bacterial taxa to the selected functions (with a contribution of the resident or transient bacterial communities exceeding 70%) enriched in the autochthonous or allochthonous bacteria after 24 h post-feeding. In the selected functions, deg. refers to degradation.

With the aim of understanding the functional profile of these modelled bacterial associations, an inferred metagenome pathway analysis was also conducted. This functional enrichment displayed specific functions that were enriched in the resident or transient microbiomes. At KEGG level 3, we identified 147 metabolic functions shared by both communities; of these, 54 pathways were enriched in the autochthonous bacteria and 44 in the allochthonous community. We then selected functions for which either community contributed more than 70% (Figure 6a), yielding 14 pathways enriched in the resident microbiota (e.g., protein digestion and absorption, phenylalanine metabolism, vancomycin biosynthesis, polycyclic aromatic hydrocarbon degradation, sphingolipid metabolism, and N-glycan biosynthesis) and 11 in the transient microbiota (e.g., fructose and mannose metabolism, primary and secondary bile acid biosynthesis, retinol metabolism, and tetracycline biosynthesis). Additionally, the contributions of each cluster to the selected functions were represented in Figure 6b. The achieved results support the functional relevance of clusters 4 and 5 in the resident bacteria, while highlight the contribution of clusters 2 and 7 to the pathways enriched in the allochthonous microbiome.

## 4. Discussion

There is increasing evidence for the crucial role of the intestinal microbiome in maintaining host health within aquaculture systems [53]. Consequently, in recent decades a significant growth in studies on fish microbiomes has been reported [4]. In this regard, several factors must be considered when designing such studies, including the target bacterial group (autochthonous and/or allochthonous bacterial communities), the sampling site (intestinal section), and the post-feeding sampling time [54,55]. These parameters may be species-dependent, since variations in intestinal anatomy and morphology associated with distinct dietary habits (carnivorous, herbivorous, or omnivorous) influence the structural integrity and fragility of intestinal tissues [32]. Nevertheless, methodological aspects related to intestinal sampling remain non-standardized. For example, among microbiome studies on gilthead sea bream conducted over the past eight years, some have focused exclusively on the resident or transient bacterial community, while others have examined both fractions either separately or together [17,18,25,27,28,56,57,58,59,60,61,62,63,64,65,66,67,68,69,70]. In the present study, we investigated the differences between autochthonous and allochthonous bacterial communities in the anterior and posterior intestinal sections of gilthead sea bream at 24 h post-feeding, as well as the differences between autochthonous microorganisms in both intestinal sections at 24 and 48 h post-feeding. Both bacterial communities exhibited marked differences in structure and composition. However, spatial differences across the intestine were less pronounced, especially in the case of autochthonous bacteria, being even lower the fluctuations over feeding time for the resident community. Furthermore, the use of Bayesian network approach allowed to identify interactions among bacteria colonizing both communities, revealing numerous causal relationships within these populations. In addition, the inferred functional enrichment displayed numerous shared KEGG metabolic pathways at level 3 between both types of bacteria, although each community showed enrichment in distinct and physiologically relevant functions.

As previously reported in Atlantic salmon [34] and rainbow trout [71,72,73], the transient community often presents greater values of richness and diversity indices than the resident bacteria. These results are consistent with previous observations in gilthead sea bream [64], corroborating the patterns identified in the present study (Figure 1 and Table S1). This increased diversity may result from the mixing of environmental microbes and those from the diet with the digesta [23,24]. Accordingly, our observations, consistent with previous studies [74,75], suggest that the mucus layer provides a more restrictive environment, supporting the adherence and persistence of a specialized and stable microbial community. In line with this notion, strong differences in composition between resident and transient microbial communities were detected regardless of the sampled intestinal region (Figure 2). At the phylum level, Pseudomonadota (formerly Proteobacteria) was predominant in the resident community, while Bacillota (formerly Firmicutes) represented the most abundant taxa in the transient bacteria, regardless of the sampling section and post-feeding time (Figure 2a and Figure 3c). Indeed, these phyla have also been identified as the most prevalent in previous fish microbiome studies, either in other fish species (European sea bass and/or rainbow trout [29,72,73,76]) or in gilthead sea bream [5,13,16,17,63,77,78,79,80,81]. Certainly, Bacillota has been suggested to exhibit a more transient nature than other phyla [5], being considered the most abundant phylum within the allochthonous bacterial community of the distal intestine in gilthead sea bream [77,78]. Furthermore, as previously reported [64], the genus *Lactobacillus*, belonging to the Bacillota phylum, was the most represented taxa in the transient bacteria. These microorganisms may come from the feed, which is consistent with the reported greater effect of the feed bacteria on the transient community [13,23]. Indeed, most Bacillota microbes detected here, such as *Lactobacillus* or *Clostridium*, are facultative or strict anaerobes, whereas members of Pseudomonadota identified in the intestinal mucus are often able to tolerate a range of oxic conditions. In this regard, the fish gut environment typically has higher oxygen levels than in mammals [2], and in wild or fasted farmed fish the intestines are likely not permanently oxygen depleted [13]. Thus, this context might favour the establishment of Pseudomonadota in the mucus layer rather than Bacillota species. Moreover, it has been reported that *Vibrio* species, which belong to the phylum Pseudomonadota, have the fastest growth rates among bacteria [82], reinforcing the putative favourable conditions for these microorganisms to proliferate in the intestinal mucus. Indeed, the main taxa in the resident community belonged to the genera *Mesorhizobium* and *Aliivibrio*, of the phylum Pseudomonadota, which are aerobic or facultative aerobic species largely identified in the gut microbiome of several fish species [83,84,85,86,87], including gilthead sea bream [20,35,60].

Regarding post-prandial time, the discriminant analysis exhibited no clear effects of the post-feeding time (24 and 48 h) on the autochthonous microbiome. In contrast, a longer period after feeding (86 h) induced remarkable shifts in the gut microbiome composition of gilthead sea bream [13], which might be due to the effects of relevant physiological and molecular changes (e.g., oxidative stress) promoted in the intestinal tissue after several days of fasting [88,89]. Therefore, as stated by Navarro-Guillen and Yufera [90], the fish gut microbiome is clearly influenced by the time elapsed since the last feeding, with major changes occurring in the gut microbiome during the first 6 h after feeding [91,92]. Indeed, fluctuations of gut microbiota may be more subtle at later stages, particularly at low water temperatures (≈16 °C on our sampling date), as evacuation rates decrease under cooler conditions [54,93,94,95], making the delayed gut transit time a key factor in shaping the fish bacterial composition [92]. On the other hand, we found herein that the spatial changes in the transient microbial community were minimal and mainly attributed to changes in low abundant bacterial groups (Figure S2). Accordingly, no differences were found in the autochthonous microbiomes of Atlantic salmon [34] or in both resident and transient communities of gilthead sea bream [13] along the spatial gut. The lack of microbiota divergence along the intestine in a carnivorous species such as gilthead sea bream might be due to the shorter digestive tract in comparison to those of herbivores or omnivores [32]. However, there is not a clear pattern across fish species because spatial divergences in microbial gut communities have been reported in carnivorous fish such as Atlantic salmon [9] and European sea bass [12], with the inclusion of the pyloric caeca as part of the analyzed tissue portion in the former or the increase in the evacuation rate with the rise in the water temperature (25 °C) in the latter. In this regard, it appears that, in the present study, low water temperatures likely slowed the transit rate and hindered the detection of potential differences in microbiota along the intestine. Conversely, strong differences were detected, in terms of composition, between resident and transient bacterial communities, regardless of the sampled intestinal region (Figure 3), in agreement with previous studies on different fish species [30,54], including gilthead sea bream [64]. This contrasts with the lack of differences observed in gilthead sea bream at 3 h post-feeding [13], which might be explained by the different sampling time and/or the important role of feeding in shaping fish gut microbiome composition [92].

Following the previous observations, the Bayesian network was built with samples of the resident and transient communities collected 24 h after feeding along the intestine. Previous studies have applied co-occurrence networks to examine gut bacteria interactions and correlations [96,97], although the BN employed here also allows us to investigate the causal relationships between microbiomes and the host, establishing the hierarchy and cooperation among bacteria within a microbial community [49]. In the present study, this analysis showed that most relevant identified bacteria, except *Synechococcus* (Figure 4), were connected, directly or indirectly, to the variable type of bacteria, and only around 9–11% of the intestinal microbiota displayed no correlation with the mentioned variable (Figure 5). Remarkably, most bacteria were present in both communities and there were numerous interconnections between microbes with a significantly greater abundance in a different community, suggesting feasible interactions between resident and transient communities, as previously reported in humans [98,99]. In addition, the inferred metagenomic functional enrichment allowed to identify numerous putative overlapping KEGG pathways that were common to the two communities (i.e., there were bacteria performing many functions in both), which might be expected based on the fact that most microorganisms were identified in both resident and transient communities and on previous studies showing functional redundancy between them [64]. These findings support the notion that bacteria from both communities interact synergistically to influence host-associated functions. Furthermore, besides this cooperation between both resident and transient microorganisms, several inferred pathways were potentially enriched in autochthonous or allochthonous communities. At this point, it should be remarked that, despite the use of PICRUSt2 with 16S rRNA gene amplicon sequencing data may have limitations and its inferences must be interpreted with caution, this tool represents a useful available strategy to assess putative metabolic pathways in non-human microbiomes. In the present study, a stringent filter was applied and only those functions for which the contribution of resident or transient bacteria exceeded 70% were highlighted (Figure 6a). Thus, among the selected functions, we will focus mostly on pathways linked with metabolism. Resident bacteria appear to be primarily associated with protein digestion and absorption, as well as phenylalanine metabolism, pathways related to protein metabolism. Indeed, *Polaribacter* strains of marine origin are able to produce peptidases [100], and an increase in *Brevundimonas* abundance has been associated with enhanced intestinal digestion and absorption in eel [101]. In this regard, protein is the most abundant nutrient in aquaculture fish feeds and a critical component for growth. Consequently, protein is expected to be the predominant feed-derived compound reaching the intestine, and the established resident bacteria are likely the main microorganisms responsible for processing these substrates. Concerning the sphingolipid metabolism, it has been reported that Bacteroidota species, such as those of the genus *Polaribacter*, may produce compounds that have an active role in maintaining the host sphingolipid homeostasis [102]. On the other hand, N-glycan biosynthesis occurs in some bacteria, and it involves the N-glycosilation, i.e., the attachment of sugar molecules to asparagine residues in proteins [103]. This pathway may be important for survival and adhesion [104,105] and could potentially help some resident bacteria detected in this study (e.g., *Brevundimonas*, *Mesorhizobium*, *Aliivibrio*, *Polaribacter*) to adhere to the gut mucus.

Several bacteria presenting significantly greater abundances in the allochthonous community have been previously considered as potential probiotics in aquaculture (e.g., *Lactobacillus*, *Bacillus*, *Staphylococcus*, *Pediococcus*, *Clostridium*, *Oceanobacillus* [106,107,108]). In this sense, the formation of antimicrobial compounds has often been reported in several bacteria, including probiotics [106,109,110], and it is plausible that some of the mentioned microorganisms might play a role in enhancing tetracycline biosynthesis within the transient community, as a mechanism to enhance their ability to create a favourable environment. At this point, it is noteworthy that the function related to biosynthesis of vancomycin group antibiotics may also appear in some resident bacteria, such as *Escherichia*-*Shigella* [111]. Resuming the discussion on the putative pathways enriched in the allochthonous microbial community, several transient bacteria (e.g., *Lactobacillus*, *Staphylococcus*, *Pediococcus*) seem to be potentially involved in functions related to carbohydrate metabolism (i.e., fructose and mannose metabolism and PTS). PTS is utilized by bacteria to take up extracellular sugars, and, for instance, *Staphylococcus* bacteria have been correlated with this system, with PTS-related metabolic genes particularly active in cellobiose and mannose metabolism [112]. In this regard, we may speculate that due to the transient nature of these bacteria, they have specialized on carbohydrate metabolism, as this provides them with energy faster than other macronutrients [113]. Moreover, metabolic pathways potentially associated with some of these transient microorganisms appear to be beneficial to the host, such as primary and secondary bile acid synthesis, related to lipid metabolism and considered part of the bacteria–host associations by regulating the immune system and improving the lipid digestion [114,115]. Certainly, a number of different gut microbial taxa have been reported to be able to transform bile salts/acids [116], including members of the genera *Lactobacillus* and *Clostridium*. These putative beneficial effects support the idea that some taxa detected at higher abundance in the transient community (e.g., *Lactobacillus*, *Staphylococcus*, *Pediococcus*) could be considered as potential probiotics. Certainly, these putative beneficial strains do not necessarily colonize the mucus layer to exert positive responses [38,39] and can have an impact forming part of the transient microbiome, either directly by their own metabolic activity or alternatively through their interaction with autochthonous bacteria, as suggested in this work. Overall, our results support the notion that, in the fish intestine, autochthonous and allochthonous microorganisms likely maintain a synergistic relationship that may benefit the host by aiding several metabolic functions. Resident bacteria provide a stable community and are predicted to contribute primarily to protein digestion and metabolism. Complementarily, the transient community seems to assist in carbohydrate and lipid metabolism and includes taxa previously used as probiotics in aquaculture [106,108], which may confer benefits by producing beneficial compounds such as bile salts.

## 5. Conclusions

**Figure 7:**
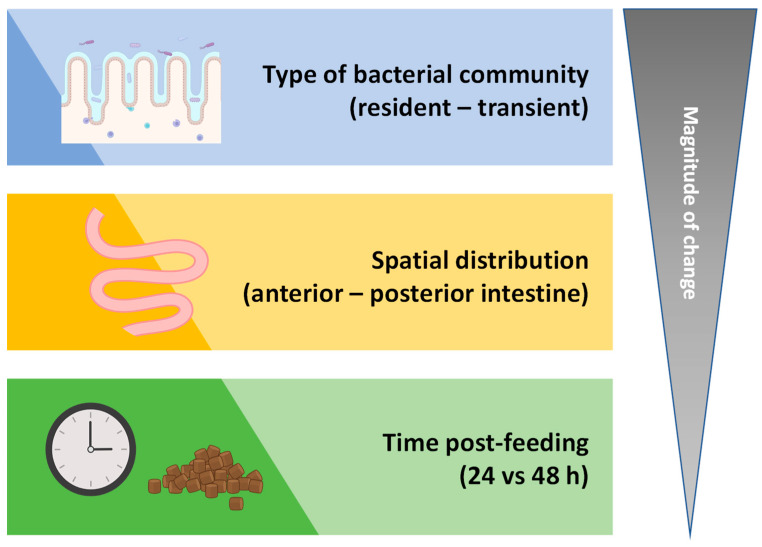
Schematic representation of the strength of the effects of the three elements influencing the gut microbiome that were investigated in this work. Icons created with Biorender.com.

This study demonstrates that the type of bacterial community (autochthonous vs. allochthonous) is the main driver shaping gut microbiome composition in gilthead sea bream, outweighing the effects of intestinal location and post-feeding sampling time within the tested window (Figure 7). Autochthonous and allochthonous microbiota form distinct yet interconnected communities with potentially complementary functional roles, with mucus-associated bacteria likely contributing predominantly to protein-related metabolic pathways and lumen-associated bacteria to carbohydrate and bile acid metabolism. These findings emphasize the importance of explicitly targeting the appropriate gut microbial fraction according to the biological question and functional processes of interest and provide methodological guidance for future fish microbiome studies.

## Data Availability

All the basecalled data (FASTQ files) used in this work were loaded in the Sequence Read Archive (SRA) under the Bioproject accession number PRJNA1372998 (BioSample accession numbers: SAMN53634082-121).

## Supplementary Materials

The following supporting information can be downloaded at: https://www.mdpi.com/article/10.3390/ani16030360/s1, Figure S1: (a) Two-dimensional PLS-DA Score plot representing the distribution of the samples between the first two components in the model driving the separation of the resident (either from the anterior or posterior intestine, Rd-AI1 and Rd-PI1) bacteria after 24 or 48 h post-feeding. (b) Validation plot of the PLS-DA model shown in (a), consisting in 500 random permutations; Figure S2: (a) Two-dimensional PLS-DA Score plot representing the distribution of the samples between the first two components in the model driving the separation of the resident (either from the anterior or posterior intestine, Rd-AI1 + Rd-PI1) and transient (either from the anterior or posterior intestine, T-AI and T-PI) bacteria after 24 h post-feeding. (b) Validation plot of the PLS-DA model shown in (a), consisting in 500 random permutations. (c) Loading plot of the ecological grouping in the PLSDA model shown in (a) reporting the main taxa markers associated with transient bacteria, either from the anterior (AI) or posterior (PI) intestine; Figure S3: Validation plot of the PLS-DA model, consisting in 500 random permutations, of microbiome data for the resident (either from the anterior or posterior intestine, Rd-AI1 + Rd-PI1) and transient (either from the anterior or posterior intestine, T-AI and T-PI) bacteria after 24 h post-feeding; Table S1: Richness estimators (Chao1 and ACE) and diversity indices (Shannon and Simpson) for the resident (Rd) and transient (T) microbiomes of anterior (AI) and posterior (PI) intestine after 24 (1) or 48 h (2) post-feeding (n = 6–8). Different letters indicate significant differences among sample types (Kruskal–Wallis test with Dunn’s post-test, *p* < 0.05); Table S2: Relative abundances (%) of bacterial taxa (phyla, family and genera) for the resident (Rd) and transient (T) microbiomes of anterior (AI) and posterior (PI) intestine after 24 (1) or 48 h (2) post-feeding (n = 6–8). Different letters indicate significant differences among sample types (Kruskal–Wallis test with Dunn’s post-test, *p* < 0.05).
