# Supplementary material for: Autochthonous and Allochthonous Gut Microbes May Work Together: Functional Insights from Farmed Gilthead Sea Bream (Sparus aurata)
Source: Animals (Basel). 2026 Jan 23;16(3):360. doi: 10.3390/ani16030360 (PMC12896760; doi:10.3390/ani16030360)
Supplement: Supplementary file 1 [file animals-16-00360-s001.zip › Table S1.pdf]

Table S1. Richness estimators (Chao1 and ACE) and diversity indices (Shannon and Simpson) for the resident (Rd) and transient (T) microbiomes of anterior (AI) and posterior (PI) intestine after 24 (1) or 48 h (2) post-feeding (n=6-8). Different letters indicate significant differences among sample types (Kruskal-Wallis test with Dunn's post-test, $p < 0.05$).

							p-value[1]				
**Index**	**Rd-AI1**	**Rd-PI1**	**T-AI**	**T-PI**	**Rd-AI2**	**Rd-PI2**	**Global**	**Type**	**Section-Rd**	**Section-T**	**Time**
Richness											
Chao1	71.03^{c}	84.90^{abc}	305.72^{a}	287.82^{a}	66.60^{c}	73.76^{bc}	<0.001	<0.001	0.24	1.00	0.286
ACE	78.95^{ab}	75.09^{ab}	286.32^{a}	275.59^{a}	59.24^{b}	74.56^{ab}	<0.001	<0.001	0.818	0.818	0.246
Diversity											
Shannon	1.37	1.11	2.38	1.79	1.79	1.37	0.05	0.006	0.589	0.065	0.178
Simpson	0.54	0.42	0.77	0.62	0.68	0.50	0.225	0.033	0.589	0.093	0.194

[1] Level of statistical significance of the Kruskal Wallis test for the comparison among all groups (**Global**) or between the type of bacterial communities (resident and transient) at 24 h post-feeding (**Type**), intestinal sections at 24 h post-feeding in the resident (**Section-Rd**) or transient (**Section-T**) bacteria, and post-feeding times (24 vs 48 h) in the autochthonous community (**Time**).
