# Supplementary material for: Autochthonous and Allochthonous Gut Microbes May Work Together: Functional Insights from Farmed Gilthead Sea Bream (Sparus aurata)
Source: Animals (Basel). 2026 Jan 23;16(3):360. doi: 10.3390/ani16030360 (PMC12896760; doi:10.3390/ani16030360)
Supplement: Supplementary file 1 [file animals-16-00360-s001.zip › Table S2.pdf]

Table S2. Relative abundances (%) of bacterial taxa (phyla, family and genera) for the resident (Rd) and transient (T) microbiomes of anterior (AI) and posterior (PI) intestine after 24 (1) or 48 h (2) post-feeding (n=6-8). Different letters indicate significant differences among sample types (Kruskal-Wallis test with Dunn's post-test, $p < 0.05$).

							p-value[1]				
**Taxa**	**Rd-AI1**	**Rd-PI1**	**T-AI**	**T-PI**	**Rd-AI2**	**Rd-PI2**	**Global**	**Type**	**Section-Rd**	**Section-T**	**Time**
Phyla											
Pseudomonadota	72.52^{ab}	55.00^{ab}	15.06^{ab}	10.81^{b}	80.25^{a}	46.63^{ab}	0.005	0.001	0.699	0.818	0.610
Bacillota	6.01^{c}	8.20^{bc}	79.51^{ab}	85.00^{a}	9.08^{abc}	43.57^{abc}	<0.001	<0.001	0.818	0.485	0.057
Spirochaetota	7.64^{ab}	16.86^{ab}	0.01^{b}	0.01^{b}	2.40^{a}	6.10^{ab}	0.003	0.013	0.589	0.699	0.516
Cyanobacteria	10.84	5.12	4.28	3.63	3.82	0.66	0.035[2]	0.021	0.240	0.589	0.164
Bacteroidota	1.31	13.31	0.08	0.11	1.08	0.36	0.096	0.419	0.818	0.589	0.265
Actinomycetota	1.28	1.35	0.33	0.22	2.95	2.40	0.06	0.119	0.132	0.240	0.246
Family											
Vibrionaceae	43.27	41.03	9.79	10.00	7.18	4.10	0.073	0.033	0.589	0.485	0.007
Rhizobiaceae	16.30^{abc}	7.39^{abc}	0.21^{bc}	0.03^{c}	43.18^{a}	25.85^{ab}	<0.001	<0.001	0.240	0.065	0.014
Lactobacillaceae	3.81^{bc}	5.00^{bc}	42.51^{ab}	56.45^{a}	1.72^{abc}	0.74^{c}	<0.001	<0.001	0.937	0.132	0.926
Clostridiaceae	0.37^{b}	1.54^{ab}	4.92^{ab}	4.73^{ab}	5.11^{ab}	41.15^{a}	0.005	0.001	0.485	0.589	0.003
Spirochaetaceae	7.64^{ab}	16.86^{ab}	0.01^{b}	0.01^{b}	2.40^{a}	6.10^{ab}	0.003	0.013	0.589	0.699	0.516
Alcaligenaceae	4.23^{ab}	2.99^{ab}	0.00^{b}	0.00^{b}	10.05^{a}	10.24^{a}	<0.001	<0.001	1.00	0.937	0.012
Bacillaceae	0.21^{b}	0.28^{b}	13.21^{a}	11.25^{a}	0.56^{ab}	0.17^{b}	<0.001	<0.001	0.699	0.699	0.246
Flavobacteriaceae	1.28^{ab}	13.22^{ab}	0.03^{ab}	0.01^{b}	0.24^{a}	0.35^{ab}	0.033	0.083	0.937	0.240	0.171
Cardiobacteriaceae	0.00	0.00^{abc}	0.01	0.00	8.20	0.01	0.708	0.745	0.699	0.394	0.923
Caulobacteraceae	1.68^{abc}	0.85^{abc}	0.01^{bc}	0.00^{c}	3.57^{a}	2.60^{ab}	<0.001	0.002	0.818	0.818	0.041
Peptostreptococcaceae	0.03^{c}	0.04^{c}	4.48^{a}	3.28^{ab}	0.11^{bc}	0.06^{bc}	<0.001	<0.001	0.937	0.394	0.569
Staphylococcaceae	0.02^{b}	0.57^{ab}	1.34^{ab}	2.21^{a}	0.97^{ab}	0.92^{ab}	0.011	<0.001	0.065	0.180	0.725
Genera											
Lactobacillus	3.57^{bc}	4.94^{bc}	41.90^{ab}	55.54^{a}	1.70^{abc}	0.73^{c}	<0.001	<0.001	0.937	0.132	0.926
Mesorhizobium	14.34^{ab}	5.74^{ab}	0.07^{b}	0.01^{b}	41.33^{a}	23.32^{a}	<0.001	<0.001	0.394	0.699	0.008
Aliivibrio	38.39^{a}	29.92^{a}	0.26^{ab}	0.07^{b}	6.76^{ab}	3.88^{ab}	0.003	<0.001	0.937	0.699	0.178
Photobacterium	0.41	6.43	9.37	9.83	0.30	0.14	0.108	0.644	0.180	0.394	0.150

Bacillus	0.12^{b}	0.15^{b}	8.95^{a}	8.08^{a}	0.52^{ab}	0.12^{b}	<0.001	<0.001	0.937	0.589	0.164
Polaribacter	1.28^{ab}	13.21^{ab}	0.00^{b}	0.00^{b}	0.15^{a}	0.09^{ab}	0.006	0.002	0.937	0.937	0.365
Brevundimonas	1.68^{abc}	0.85^{abc}	0.00^{bc}	0.00^{c}	3.57^{a}	2.60^{ab}	<0.001	0.001	0.818	0.589	0.041
Vibrio	4.47	4.68	0.15	0.08	0.12	0.08	0.21	0.043	0.937	0.394	0.018
Clostridium	0.08^{b}	0.02^{b}	4.15^{a}	4.06^{a}	0.17^{ab}	0.10^{b}	<0.001	<0.001	0.180	0.589	0.026
Allorhizobium[3]	1.72^{ab}	1.51^{ab}	0.00^{ab}	0.00^{b}	1.45^{ab}	2.14^{a}	0.005	0.004	0.485	0.485	0.402
Romboutsia	0.03^{bc}	0.04^{bc}	3.55^{a}	2.79^{ab}	0.11^{c}	0.06^{c}	<0.001	<0.001	0.937	0.485	0.699
Staphylococcus	0.02^{b}	0.57^{ab}	1.33^{ab}	2.15^{a}	0.97^{ab}	0.92^{ab}	0.011	<0.001	0.065	0.180	0.725
Paenibacillus	0.11^{ab}	0.06^{ab}	3.69^{a}	1.72^{a}	0.03^{b}	0.03^{b}	<0.001	<0.001	0.818	0.180	0.142
Cutibacterium	0.00^{b}	0.54^{ab}	0.12^{ab}	0.12^{ab}	2.38^{a}	1.00^{ab}	0.007	0.126	0.132	0.699	0.002
Synechococcus	0.46^{b}	0.02^{b}	2.46^{a}	0.64^{ab}	0.21^{ab}	0.17^{ab}	0.003	<0.001	0.937	0.041	0.425
Escherichia-Shigella	2.73^{a}	0.75^{ab}	0.01^{bc}	0.00^{bc}	0.18^{abc}	0.01^{c}	<0.001	<0.001	0.485	0.937	<0.001
Microbacterium	0.76	0.66	0.01	0.00	0.39	1.29	0.004[4]	0.001	0.240	0.699	0.457
Oceanobacillus	0.07^{abc}	0.09^{c}	1.91^{a}	1.39^{ab}	0.03^{c}	0.02^{bc}	<0.001	<0.001	0.065	0.394	0.406
Rhizobacter	1.30	0.00	0.00	0.00	0.01	0.00	0.339	0.071	0.818	1.00	0.182
Chloroplast	0.90	0.27	0.00	0.00	0.00	0.01	0.279	0.071	0.699	1.00	0.137
Anaerococcus	0.00	0.00	1.38	0.01	0.21	0.08	0.08	<0.001	0.699	0.589	0.034
Methylobacterium[5]	0.03	0.09	0.00	0.01	0.55	0.00	0.674	0.515	0.699	0.937	0.951
Porphyromonas	0.00	0.00	0.00	0.00	0.62	0.01	0.688	1.00	1.00	1.00	0.212

[1] Level of statistical significance of the Kruskal Wallis test for the comparison among all groups (**Global**) or between the type of bacterial communities (resident and transient) at 24 h post-feeding (**Type**), intestinal sections at 24 h post-feeding in the resident (**Section-Rd**) or transient (**Section-T**) bacteria, and post-feeding times (24 vs 48 h) in the autochthonous community (**Time**).; [2] Only a trend to a statistically significant difference between T-AI and Rd-PI1 were detected; [3] *Allorhizobium-Neorhizobium-Pararhizobium-Rhizobium*; [4] Only a trend to a statistically significant difference between Rd-AI1 and T-PI were detected; [5] *Methylobacterium*[3]*-Methylorubrum*.
