# Supplementary figures and images for: Autochthonous and Allochthonous Gut Microbes May Work Together: Functional Insights from Farmed Gilthead Sea Bream (Sparus aurata)
Source: Animals (Basel). 2026 Jan 23;16(3):360. doi: 10.3390/ani16030360 (PMC12896760; doi:10.3390/ani16030360)

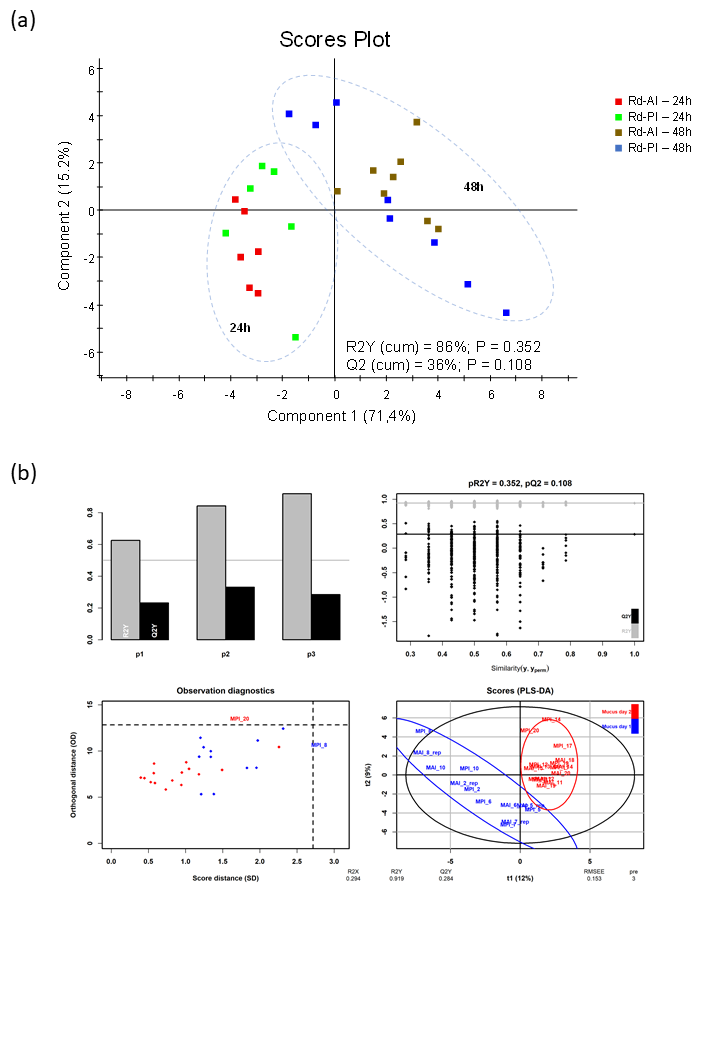

Supplement: Supplementary file 1 [file animals-16-00360-s001.zip › Figure S1.tif]

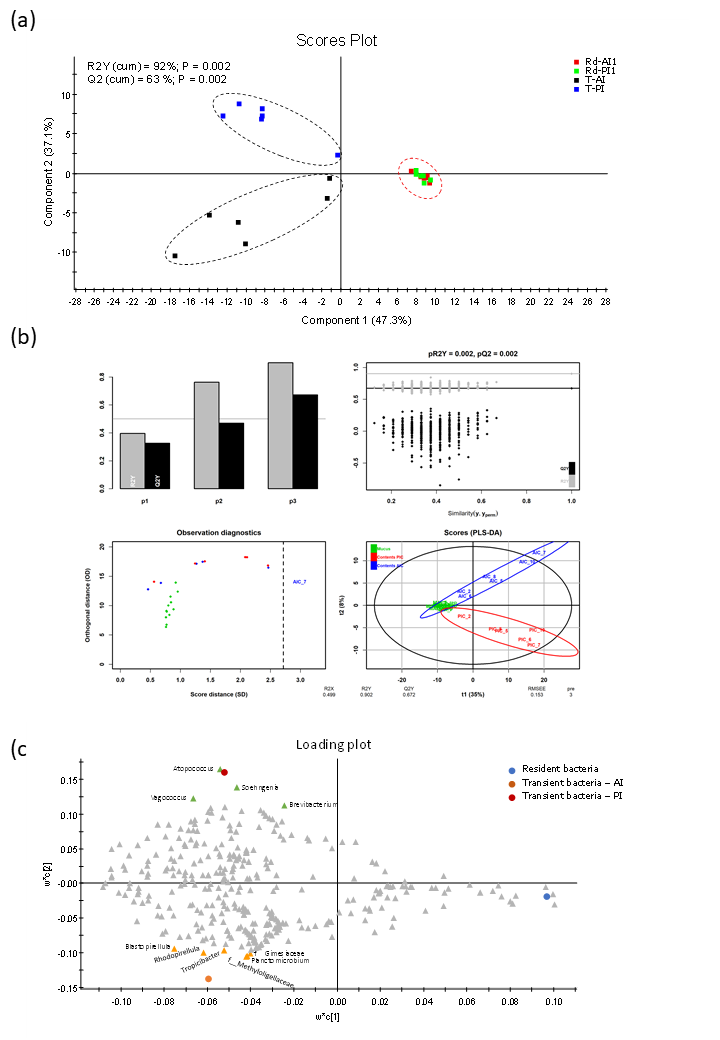

Supplement: Supplementary file 1 [file animals-16-00360-s001.zip › Figure S2-REVISED.tif]

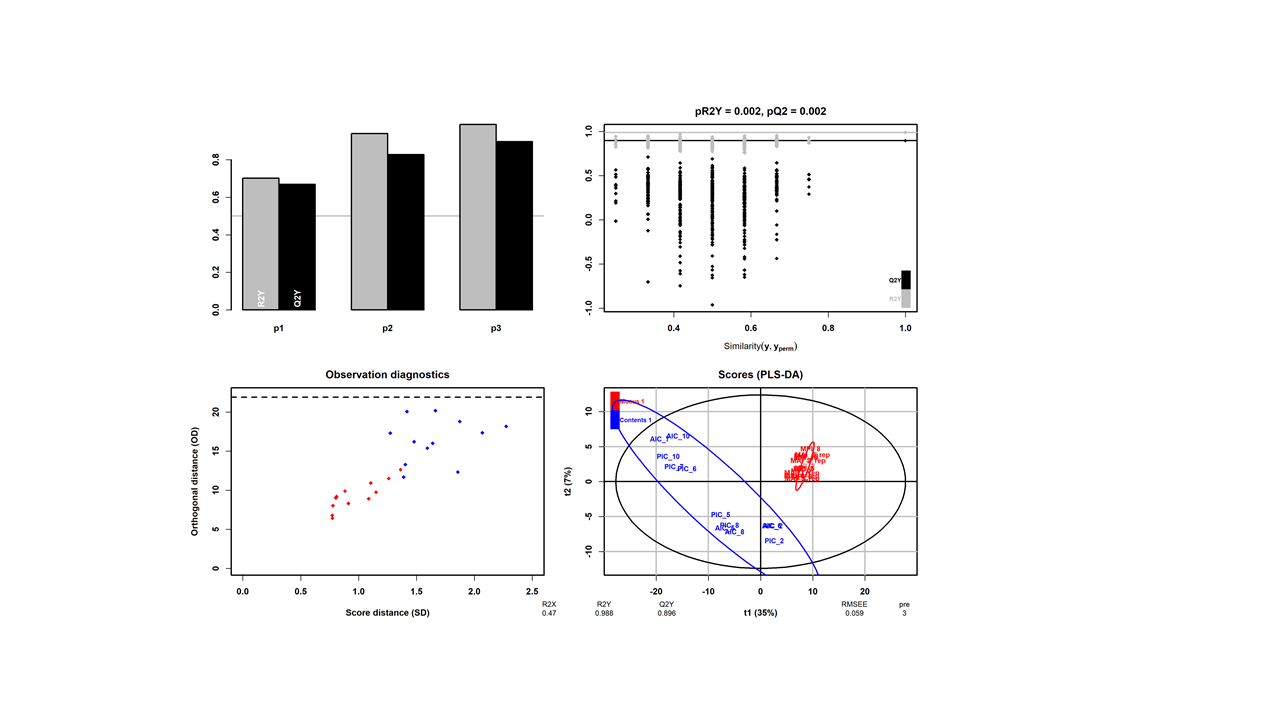

Supplement: Supplementary file 1 [file animals-16-00360-s001.zip › Figure S3.tif]
